# The impact of selective HDAC inhibitors on the transcriptome of early mouse embryos

**DOI:** 10.1186/s12864-024-10029-3

**Published:** 2024-02-05

**Authors:** Ruiqi Shao, Takayoshi Suzuki, Mikita Suyama, Yuichi Tsukada

**Affiliations:** 1 Division of Bioinformatics, Medical Institute of Bioregulation, Kyushu University, 3-1-1 Maidashi, Higashi-ku, 812-8582 Fukuoka, Japan; 2 SANKEN, Osaka University, 8-1 Mihogaoka, 567-0047 Ibaraki, Osaka Japan; 3 Advanced Biological Information Research Division, INAMORI Frontier Research Center, Kyushu University, 744 Motooka, Nishi-ku, 819-0395 Fukuoka, Japan

**Keywords:** HDAC inhibitor, MGCD0103, T247, Zygotic gene activation, Early embryos, Epigenetic reprogramming, Transcriptome

## Abstract

**Background:**

Histone acetylation, which is regulated by histone acetyltransferases (HATs) and histone deacetylases (HDACs), plays a crucial role in the control of gene expression. HDAC inhibitors (HDACi) have shown potential in cancer therapy; however, the specific roles of HDACs in early embryos remain unclear. Moreover, although some pan-HDACi have been used to maintain cellular undifferentiated states in early embryos, the specific mechanisms underlying their effects remain unknown. Thus, there remains a significant knowledge gap regarding the application of selective HDACi in early embryos.

**Results:**

To address this gap, we treated early embryos with two selective HDACi (MGCD0103 and T247). Subsequently, we collected and analyzed their transcriptome data at different developmental stages. Our findings unveiled a significant effect of HDACi treatment during the crucial 2-cell stage of zygotes, leading to a delay in embryonic development after T247 and an arrest at 2-cell stage after MGCD0103 administration. Furthermore, we elucidated the regulatory targets underlying this arrested embryonic development, which pinpointed the G2/M phase as the potential period of embryonic development arrest caused by MGCD0103. Moreover, our investigation provided a comprehensive profile of the biological processes that are affected by HDACi, with their main effects being predominantly localized in four aspects of zygotic gene activation (ZGA): RNA splicing, cell cycle regulation, autophagy, and transcription factor regulation. By exploring the transcriptional regulation and epigenetic features of the genes affected by HDACi, we made inferences regarding the potential main pathways via which HDACs affect gene expression in early embryos. Notably, Hdac7 exhibited a distinct response, highlighting its potential as a key player in early embryonic development.

**Conclusions:**

Our study conducted a comprehensive analysis of the effects of HDACi on early embryonic development at the transcriptional level. The results demonstrated that HDACi significantly affected ZGA in embryos, elucidated the distinct actions of various selective HDACi, and identified specific biological pathways and mechanisms via which these inhibitors modulated early embryonic development.

**Supplementary Information:**

The online version contains supplementary material available at 10.1186/s12864-024-10029-3.

## Background

Histone acetylation refers to the addition of an acetyl group to the ε-amino group of lysine residues located at the N-terminal tail of histones [1]. Histone acetylation is a dynamically balanced state that is regulated by the counteracting actions of histone acetyltransferases (HATs) and histone deacetylases (HDACs) [2] and contributes to the precise control of gene expression in various biological processes. In mammals, zinc-dependent classical HDACs can be categorized into three classes based on their homology to yeast counterparts: Class I (HDAC1, 2, 3, and 8), Class II (HDAC4–7 and 9–10), and Class IV (HDAC11) [3–5]. Despite being abbreviated from “histone deacetylases,” it is worth noting that HDACs (more accurately termed “lysine deacetylases”) also exhibit deacetylase activity toward non-histone proteins [6]. Specifically, only HDAC1, 2, 3, and 6 have been demonstrated to possess lysine deacetylase activity in vitro [7, 8]. Conversely, HDAC8 and HDAC11 exhibit deacylase activity toward fatty acids in vitro [9, 10]. However, the true substrates and functions of other Class II HDACs remain unclear [11].

HDAC inhibitors (HDACi) pertain to natural or synthetic small molecules that can inhibit the activity of HDACs. In disease-related applications, HDACi suppress the proliferation of multiple cancer cells or impede the transition of tumor cells toward a malignant phenotype [12]. As promising epigenetic drugs, research on the development of HDACi has been expanding, with over 30 compounds currently used in experimental studies and five approved by the FDA for clinical cancer therapy [13]. Based on their primary targeting chemical functional groups, HDACi can broadly target all types of HDACs (pan-HDACi) or specifically target a particular class or type of HDACs (selective HDACi) [4, 12, 13]. The investigations related to HDACi have greatly contributed to our understanding of the involvement of various HDACs in transcriptional activation, metabolism, DNA damage, cell cycle regulation, autophagy, angiogenesis, and other related mechanisms in the context of cancer or immune-related cells [5].

The application of HDACi in early embryos initially focused on the maintenance or restoration of cellular pluripotency and totipotency. Several studies have indicated that low doses of HDACi support the self-renewal of human and mouse embryonic stem cells and revert mouse embryonic bodies to an undifferentiated state [14, 15]. In turn, treatment with HDACi after somatic cell nuclear transfer in cloned embryos significantly improves full-term development [16, 17]. In another study, HDACi was identified as a component of a culture medium that was essential for directing induced pluripotent stem cells toward a more totipotent state, known as 8-cell-like cells [18]. The essence of this cellular conversion from a differentiated/pluripotent state to a pluripotent/totipotent state is highly likely triggered by the reprogramming of acetylation patterns induced by HDACi. Mammalian pre-implantation embryos undergo maternal-to-zygotic transition (MZT), thus acquiring totipotency and subsequently experiencing a gradual loss of this characteristic; this is accompanied by extensive remodeling of the epigenetic landscape, including histone modifications [19–21]. The methylation of histones has been extensively studied; however, recent research has shed light on the dynamic changes in acetylation of histones after fertilization, which highlights its crucial role in the regulation of zygotic gene activation (ZGA) during MZT [22]. Pan-HDACi, such as TSA or SAHA, have been used to induce the hyperacetylation of histones in studies of early embryos [22–25]. Nevertheless, the use of selective HDACi has been scarcely explored.

Therefore, we conducted a comprehensive investigation of two selective HDACi, namely MGCD0103 targeting mainly Class I HDACs (HDAC 1, 2, 3) [26] and T247 (targeting HDAC3 exclusively) [27], to examine their effects on the transcriptome of early embryos. This study provides possibilities for the application of selective HDACi in early embryos, thereby deepening our understanding of the distinct role of Class I HDACs in the epigenetic reprogramming of early embryos and expanding the potential applications of HDACi in reproductive medicine.

## Results

### Global analysis of the effects of HDACi treatment on early embryos

**Fig. 1:**
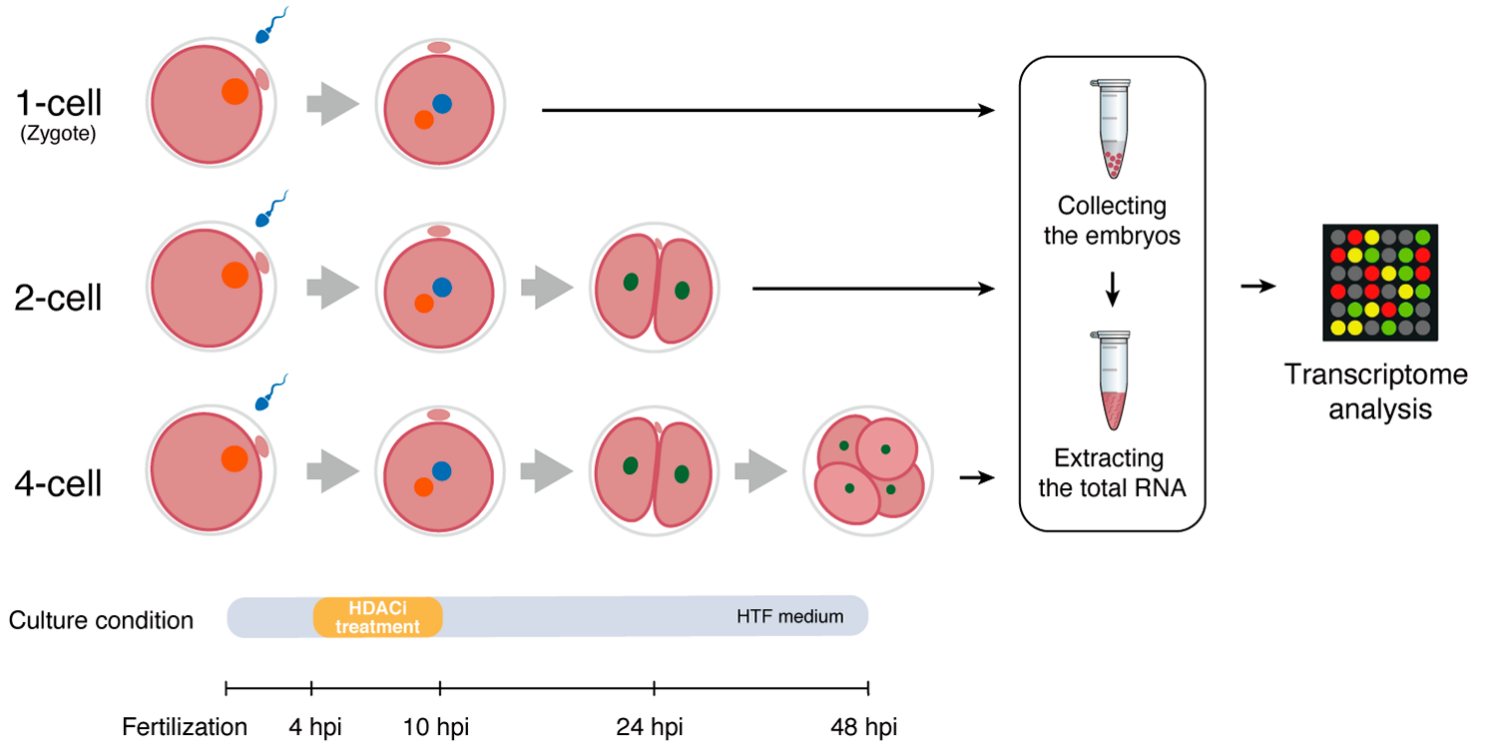
Schematic overview of experiment. Schematic showing the preparation of mouse embryos treated with HDACi for transcriptome analysis. Each sample consisted of 100 embryos, and experiment was repeated with three independent samples (biological replicates)

To explore the effects and underlying mechanisms of Class I HDACs on early embryonic development at the transcriptome level, we designed an experiment in which, after in vitro fertilization, normal embryos were treated in two culture media containing an HDACi: i.e., the T247 and MGCD0103 inhibitors, respectively. Subsequently, the embryos at the zygote stage were transferred back to the normal medium for further culture, and total RNA was extracted from embryos that developed to the 1-cell, 2-cell, and 4-cell stages, for microarray analysis (Fig. 1).

**Fig. 2:**
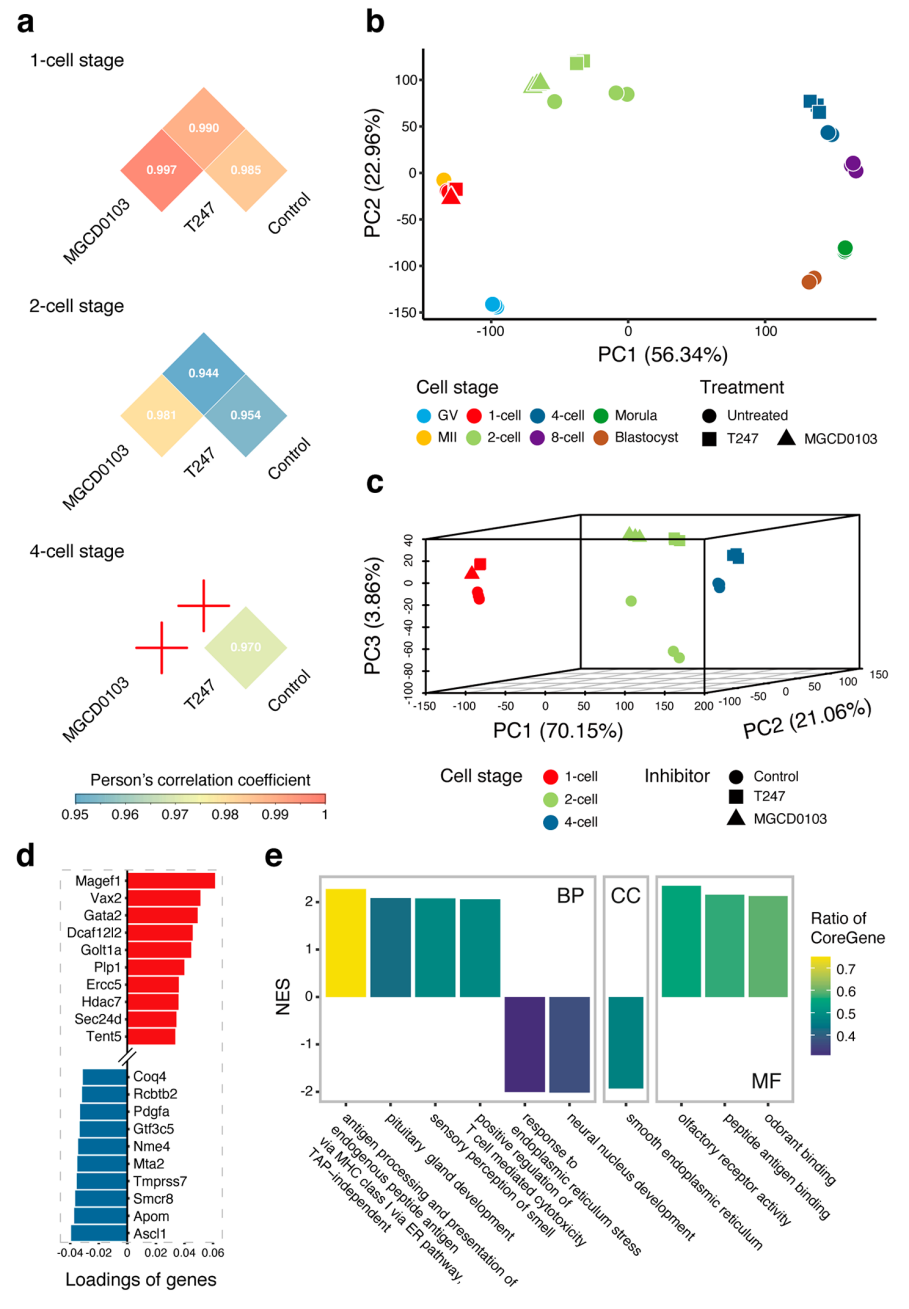
Outline of the effects of HDACi on early embryos. (**a**) Heatmap of the correlation between the HDAC inhibitors (HDACi) treatment and the control groups at three developmental stages. The mean of Pearson’s correlation coefficients of replicate samples within the treatment is shown. The red crosses indicate embryos that failed to develop to the corresponding stage after HDACi treatment. Results of the principal component analysis of combined data from other normal pre-implantation embryos (**b**) and of data from the HDACi treatment and control groups alone (**c**). The colors indicate the different developmental stages, and the shapes indicate the different treatment conditions. (**d**) The bar graph shows the genes with the highest loadings in the PC3 direction in (c), where red indicates a positive correlation between gene expression and the HDACi effects, and blue indicates a negative correlation. (**e**) Bar graph showing the 10 gene ontology (GO) terms with the highest absolute NES values in the enrichment analysis, using the loading order of genes in the PC3 direction in (c). NES: normalized enrichment score; CoreGene: genes in the ranked gene list before (for positive ES) or after (for negative ES) the peak in the running enrichment score

The results of the microarray analysis revealed a more noticeable difference between the stages of embryonic development (sample correlation coefficient range, 0.53–0.70) compared with the effect of the HDACi treatment (sample correlation coefficient range, 0.97–0.99) (Fig. S1). Therefore, by analyzing the effect of HDACi treatment using developmental stage grouping, we were able to observe that the data displayed peak-like fluctuations as the embryonic development progressed: HDACi effects were minimal at the 1-cell stage, became strong at the 2-cell stage, and decreased upon entering the 4-cell stage; moreover, MGCD0103 yielded greater differences vs. the control compared with T247 at the 2-cell stage, and, more importantly, embryos treated with MGCD0103 were unable to develop into the 4-cell stage (Fig. 2a). For further investigation of the effect of HDACi on embryonic development, we integrated the data with normal germinal vesicle (GV) stage oocytes, Metaphase II (MII) stage oocytes, 8-cell, morula, and blastocyst data, for principal component analysis (PCA) (Fig. 2b). The results of the PCA revealed that normal pre-implantation data from the GV stage to the blastocyst stage formed a directed circular developmental trajectory consistent with previous reports [28]. Starting from the 2-cell stage, the relative positions of the HDACi-treated samples compared with the control samples were all in the opposite direction of embryonic development. One possible implication of this finding is that HDACi treatment delays the development of early embryos. This effect occurs primarily at the 2-cell stage with varying degrees of effect power, depending on the type of HDACs that were inhibited: T247 may cause a development delay, whereas MGCD0103 can completely arrest embryonic development.

Subsequently, to illustrate in greater detail the differences among the treated samples, we performed a PCA only on the HDACi treatment and control data, and selected the first three principal components for visualization. After dimensionality reduction, the analysis separated the HDACi treatment from the control group in the PC3 direction, although this explained less than 4% of the total variance (Fig. 2c). To determine how this HDACi action, which endures across the embryonic development stages, affects development, we performed a gene set enrichment analysis (GSEA) using the loadings of all genes in the PC3 direction as the input (Fig. 2d). The results of this analysis suggested that 30 gene ontology (GO) terms were significantly enriched in the GO database (Table S1). Among them, the expression of genes detected after HDACi treatment was primarily positively correlated with “antigen processing and presentation of endogenous peptide antigen via MHC class I via ER pathway, TAP-independent,” whereas a primarily negative correlation with “neural nucleus development” was noted (Fig. 2e).

### HDACi effects on the 1-cell stage embryos

To investigate the effect of HDACi treatment on the specific developmental stages of early embryos, we subdivided ZGA into two stages, minor ZGA and major ZGA, based on previous studies [19, 29, 30] and, then analyzed them separately. The 1-cell and 2-cell stage data from this study were used for analysis corresponding to these two ZGA stages, respectively.

**Fig. 3:**
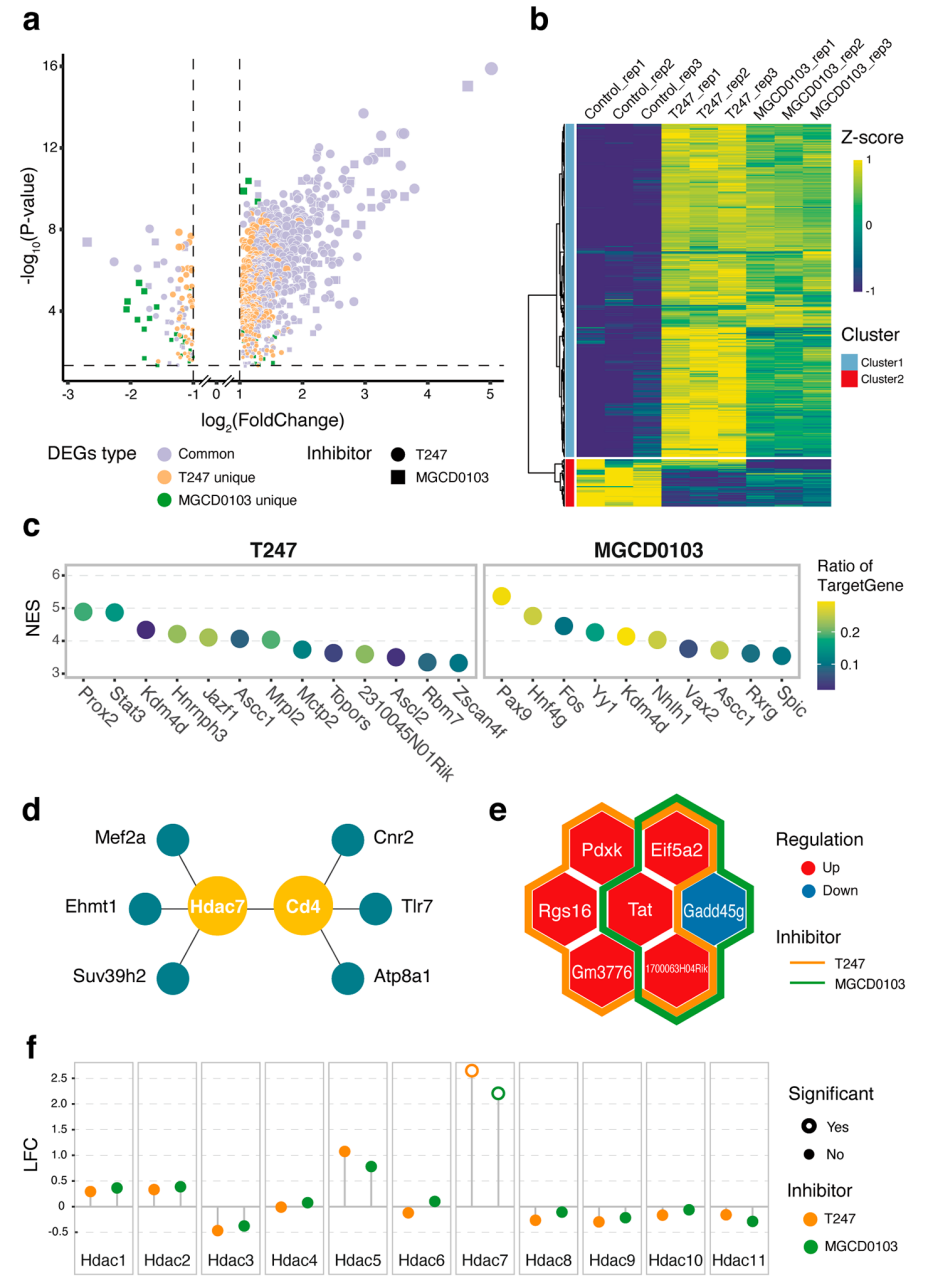
HDACi effects on 1-cell stage embryos. (**a**) Volcano plot of differentially expressed genes (DEGs), with colors representing the relationships between the DEGs in the two HDACi treatments, and point shapes indicating the HDACi treatment from which the corresponding DEGs stem. To display all data clearly, the point sizes were linearly scaled along the slopes using the same absolute values on both the positive and negative x-axes. (**b**) Heatmap of DEGs prepared using the hierarchical clustering method to divide the differential genes into two clusters. (**c**) Results of predicted transcription factors (TFs) that regulated DEGs, ranked in descending order of NES, with the color representing the ratio of DEGs among the gene set that was regulated by the corresponding TFs. (**d**) protein–protein interaction (PPI) network of HDACi-affected minor zygotic gene activation (ZGA) genes, with yellow nodes representing core proteins. (**e**) Hexagonal Venn diagram showing the presence of DEGs in the strictly filtered minor ZGA gene set. The color of the frame indicates the type of HDACi, and the genes within the frame represent the minor ZGA genes that were affected by the corresponding HDACi treatment. (**f**) Differential expression results of HDAC-family genes after HDACi treatment, where the open circles represent adjusted *P*-values < 0.05 and LFC represents the log2 fold change

To explore the direct effects on gene expression, we first identified the differentially expressed genes (DEGs) for the two HDACi treatments at the 1-cell stage. Accordingly, 677 and 433 DEGs were identified under the T247 and MGCD0103 treatment conditions, respectively (Table S2 and S3). The distribution of the DEGs for each HDACi was combined and presented in the volcano plot (Fig. 3a). Most of the DEGs (over 87%) were upregulated after HDACi treatment, indicating that the effects of HDACi on this stage occur mainly through direct action, as expected. HDACi inhibit HDACs, thus leading to a highly acetylated chromatin state, which is generally associated with gene activation [31]. Over half of the DEGs in the two HDACi treatments were shared between the two HDACi conditions (number of shared DEGs: 379); moreover, the shared DEGs predominated among the highly upregulated genes, indicating that the different HDACi-affected transcriptome expression in a similar manner or via a similar pathway at the zygote stage. We performed hierarchical clustering to investigate the existence of unique expression patterns beyond the shared genes (Fig. 3b). The results revealed two clusters that could contrast the HDACi treatments from the control, whereas no specific pattern was observed between the two HDACi.

Subsequently, we analyzed the emergent property of the DEGs identified at the 1-cell stage after HDACi treatment. Using a GO enrichment analysis, we expected to associate the DEGs with their corresponding biological information. It is noteworthy that no significantly enriched GO terms were obtained, regardless of the grouping of the data based on DEG upregulation or downregulation or the intersection relationship between the two HDACi. This is consistent with previous reports that the functional mRNAs that guide protein translation in the embryo are primarily derived from maternal deposition [21, 32], whereas transcription in the zygote itself exhibits a genome-wide feature, as it is promiscuous and uncoupled from efficient production of functional mRNAs [33]. Because HDACi affect gene expression at the transcriptional level, the DEGs identified here were the result of zygotic transcription and reflect features that are not specifically associated with any biological process. In addition, to the emergent property, we also performed an upstream regulator analysis on the sequence features for upregulated DEGs: the potential transcription factors (TFs) under the two HDACi conditions were predicted (Fig. 3c). Of note, Kdm4d was predicted as a potential regulator of DEGs in the two HDACi conditions, although the expression of Kdm4d did not change at the transcriptional level, and disappeared in the prediction of TFs in subsequent developmental stages (Fig. S2). To investigate the effect of HDACi treatment on minor ZGA, we intersected the shared DEGs with genes that are particularly expressed in zygotes compared with oocytes. Next, we used the STRING database to identify intersected genes and obtained a protein–protein interaction (PPI) network centered on Hdac7 and Cd4 (Fig. 3d). However, minor ZGA genes may differ because of variations in the experimental dataset or definition method. To explore further the effect of HDACi treatment on the actual minor ZGA process, we compared the strictly filtered 96 minor ZGA genes reported in previous studies [34], and found that only seven genes were affected by HDACi, i.e., Tat, Eif5a2, and 1700063H04Rik were upregulated by the two HDACi; whereas Gadd45g was only downregulated by MGCD0103 (Fig. 3e).

Besides the overall differential gene expression state, we also paid special attention to the expression of HDAC-family genes: only Hdac7 was significantly upregulated (LFC > 2) by the two HDACi treatments at the 1-cell stage (Fig. 3f). Moreover, this unique upregulation of Hdac7 persisted up to the 4-cell stage, which was clearly distinct from the expression patterns observed for other HDAC-family genes (Fig. S3).

### The main effects of HDACi treatment occurred at the 2-cell stage

**Fig. 4:**
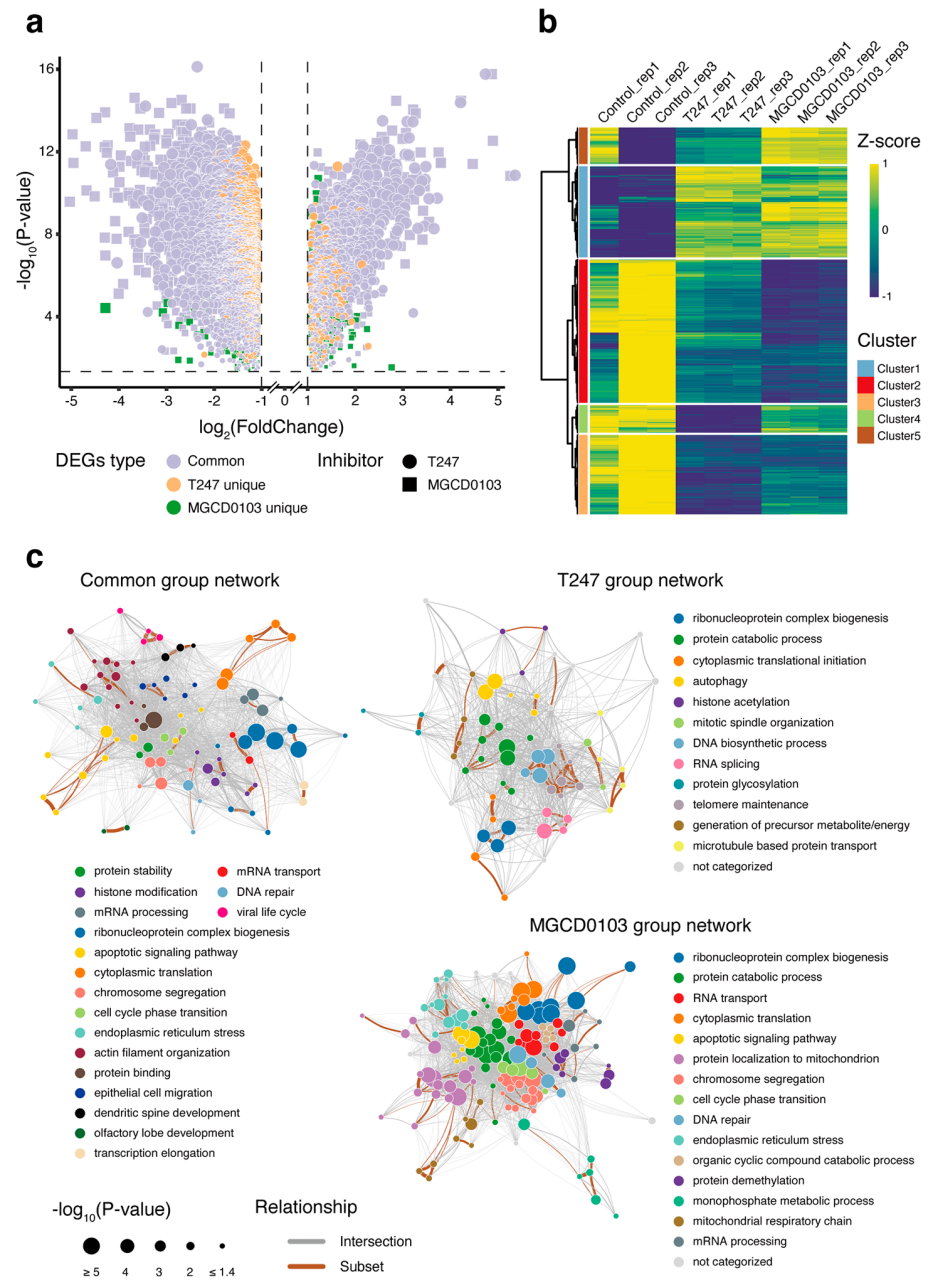
Profile of the HDACi effects on 2-cell stage embryos. (**a**) Volcano plot of DEGs, as in Fig. 3a. (**b**) Heatmap of DEGs prepared using the hierarchical clustering method, to divide the differential genes into five clusters. (**c**) The network diagram represents the enriched biological processes within each group of DEGs and their relationship. Each point represents a GO term that has been enriched, with its size indicating the negative logarithm of the *P*-value obtained from the enrichment analysis. Different colors represent different GO-term clusters, with each cluster being annotated with a label, and clusters with the same label being represented by the same color in the networks of the different groups. The width of the lines connecting the points represents the strength of the relationships between the GO terms, with brown lines connecting a pair of GO terms in which one is a subset of genes contained in the other. The layout of the network indicates that GO terms with a higher degree of correlation tended to cluster together

In the 2-cell stage data, which corresponds to the period of major ZGA, we identified 2865 and 3130 DEGs under the T247 and MGCD0103 conditions, respectively (Fig. 4a, Table S4 and S5). Compared with the 1-cell stage, the shared DEGs between the two HDACi conditions still predominated; however, most DEGs in the 2-cell stage (over 66%) were downregulated. Subsequently, we performed hierarchical clustering on all DEGs and obtained five clusters (Fig. 4b). Based on the expression pattern of each cluster, we defined clusters 1 and 3 as the “common group” under the two HDACi treatments, cluster 4 as the “T247 group,” and clusters 2 and 5 as the “MGCD0103 group.” In the subsequent analysis, we used these three groups to investigate the similarities and differences in the effects of the two HDACi at the 2-cell stage.

By the time of recovery at the 2-cell stage, the HDACi-treated embryos had already initiated major ZGA. In addition, to its direct effects, the impact of HDACi treatment is also reflected in the expanding indirect effects, thus making it challenging to pinpoint specific genes among thousands of DEGs. Here, to obtain the complete profile of the biological processes indicated by the DEGs, we developed a network representation based on the results of the GO enrichment analysis that could intuitively and comprehensively depict the inter-relationships among the enriched biological processes in the target gene set. Subsequently, we applied this procedure to each of the three groups (Fig. 4c, Table S6, S7 and S8).

Overall, the network of enriched GO terms in the MGCD0103 group was closer in distance than that observed for the other groups, indicating a stronger correlation and demonstrating common functional patterns among different biological processes. The “protein catabolic process” cluster had the highest network connectivity, indicating that the genes contained within it are widely involved in various biological processes. Moreover, clusters corresponding to cellular responses under stress conditions, such as “endoplasmic reticulum stress” and “apoptotic signaling pathway,” were connected to the “chromosome segregation,” “cell cycle phase transition,” and “DNA repair” clusters, which were related to cell cycle/division through the “protein catabolic process” cluster. This observation appears to delineate the most direct regulatory pathways that early embryos undergo, from their initial response to the perturbed environment containing MGCD0103, to their eventual developmental arrest. As observed above (Fig. 2a, b), the clusters “chromosome segregation” and “cell cycle phase transition,” which are related to cell cycle/division, were also observed in the common group representing the common effects of the inhibition of HDAC3 and Class I HDACs, whereas the T247 group, which represented specific effects of the inhibition of HDAC3 alone, was uniquely localized to the “mitotic spindle organization” cluster in terms of processes related to cell cycle/division.

### Analysis of the PPI network in the 2-cell stage embryos

The expression features of DEGs in the MGCD0103 group were consistent with the results of the experiment: MGCD0103 treatment yielded greater differences vs. the control than did T247 treatment (Fig. 4b). Furthermore, in the MGCD0103 group, a more pronounced clustering of clusters related to cell cycle/chromosome segregation was observed compared with the common group (Fig. 4c). Therefore, we hypothesized that the expression of specific functional genes in the MGCD0103 group can explain the loss of division capability observed in early embryos after MGCD0103 treatment. Based on this hypothesis, we used the STRING database to construct PPI networks for further analysis.

**Fig. 5:**
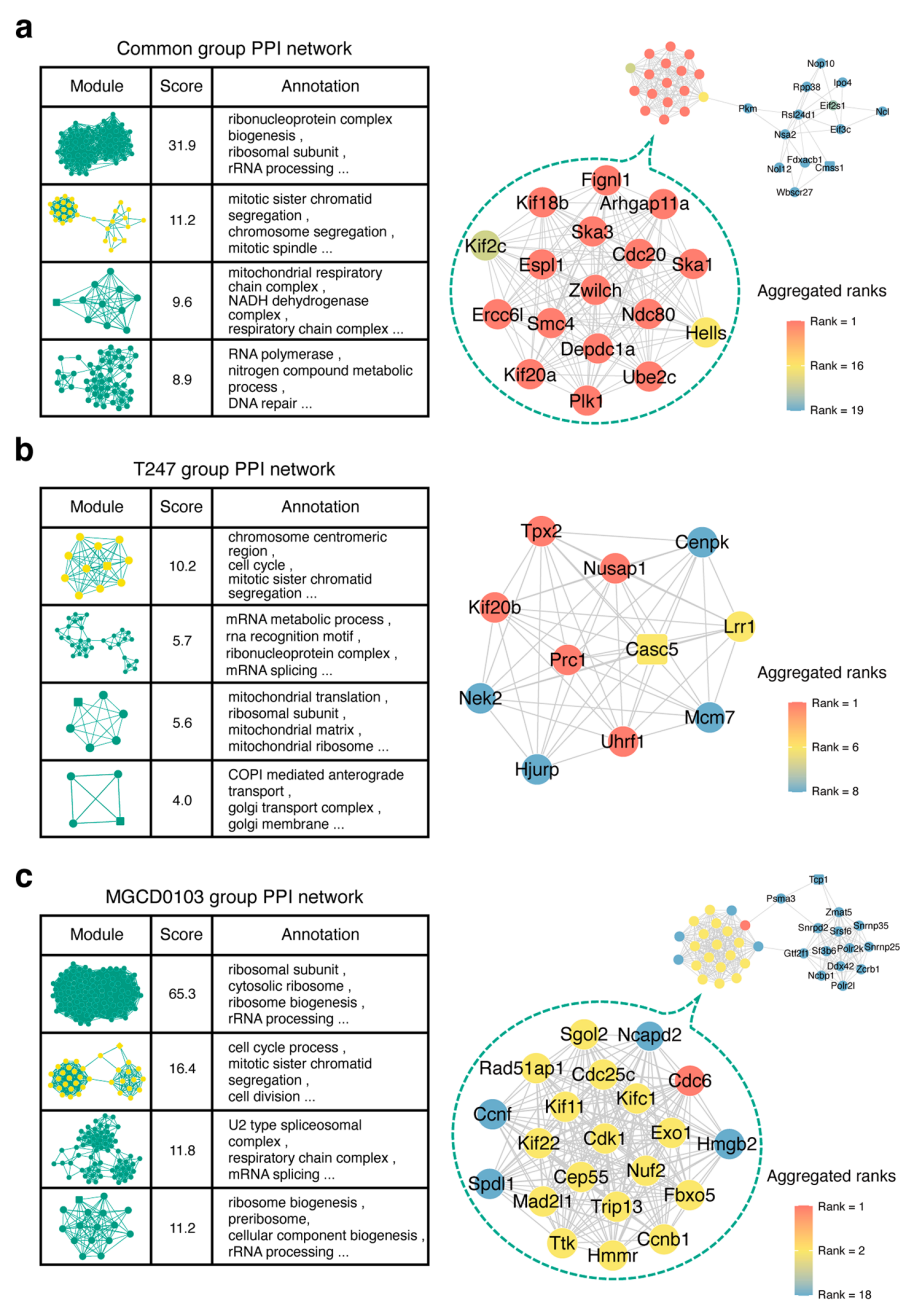
Analysis of the PPI network at the 2-cell stage. (**a**) A table listing the network structures, specific scores, and functional annotations of the top four modules with the highest scores from the complete PPI network in the DEGs of the common group is shown on the left. The details of the module highlighted in yellow are shown on the right: the protein-coding gene symbols corresponding to each node in the module are provided, with the color indicating the comprehensive ranking of the inferred importance of each node in the network (ties in the ranking are allowed). Results of the PPI network analysis in the DEGs of the T247 (**b**) and MGCD0103 (**c**) groups using the same display approach as in (a)

The overall PPI network of each group was voluminous and structurally complex; therefore, we used the MCODE plugin to extract core modules from each PPI network, for detailed analysis. Consistent with our previous observation (Figs. 2b and 4c), the common group (Fig. 5a), T247 group (Fig. 5b), and MGCD0103 group (Fig. 5c) all exhibited modules that were annotated as being related to cell cycle/chromosome segregation. In addition, the cell cycle/chromosome segregation module in the MGCD0103 group had a higher score (16.4) than it did in other groups (11.2 for the common group and 10.2 for the T247 group), and was identified as the top-scoring protein interaction subnetwork other than the ribosome-related protein complex (Fig. 5c). To illustrate these differences, we presented the full details of the cell cycle/chromosome segregation modules for the three groups in Fig. 5 (right). We also used cytoHubba to rank the importance of each gene in the protein interaction subnetwork and identified multiple genes, represented by Cdc6, as the hub genes related to the cell cycle/chromosome segregation module in the MGCD0103 group (Fig. 5c, right).

### Relationship between HDACi effects at the 2-cell stage and major ZGA

**Fig. 6:**
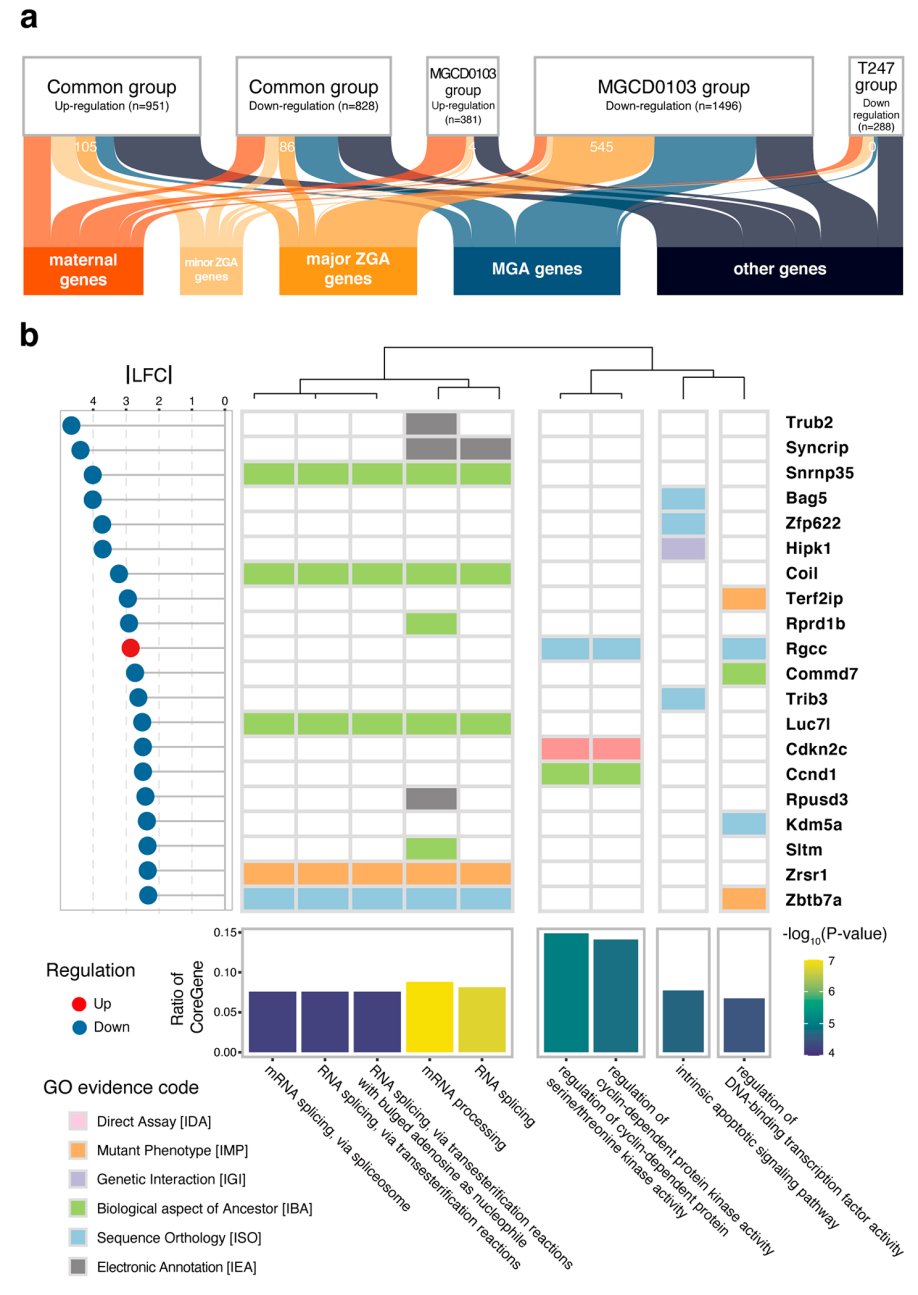
Analysis of the major ZGA genes affected by HDACi at the 2-cell stage. (**a**) The Sankey diagrams display the proportions of different expression patterns of up- and downregulated DEGs in the three groups. The upper part contains the up- and downregulated DEGs set from the three groups affected by HDACi in this experiment, whereas the lower part represents the gene sets classified according to programmed waves during maternal-to-zygotic gene activation. (**b**) The combined plot represents the presence of the top 20 major ZGA genes with the highest effect under HDACi treatment in the enriched GO terms. The main heatmap shows the presence of these 20 major ZGA genes in the GO terms that were calculated from all HDACi-affected major ZGA genes. If a gene is present in a GO term, the corresponding rectangle in the heatmap is colored based on the evidence code, which indicates how the gene is annotated in that GO term. The lollipop plot depicted on the left side of the heatmap shows the fold change in expression of these 20 genes, where|LFC| denotes the absolute value of the log2 fold change and the upregulated genes are represented in red, whereas the downregulated genes are indicated in blue. The bar chart at the bottom of the heatmap shows enriched GO terms and was divided into four clusters. The height of each bar represents the proportion of an HDACi-affected major ZGA gene in the corresponding GO term, with the color representing the negative logarithm of the *P*-value obtained from the enrichment analysis

We analyzed the relationship between the HDACi effects on the 2-cell stage embryos and major ZGA. The results showed that, in the common group, there was no significant difference in the up- and downregulation of major ZGA genes, i.e., 105 out of 951 genes and 86 out of 828 genes, respectively (Fisher’s exact test *P*-value: 0.7033), whereas in the MGCD0103 group, the proportion of major ZGA genes that were downregulated by HDACi was significantly higher than that of genes that were upregulated, i.e., 545 out of 1496 genes and four out of 381 genes, respectively (Fisher’s exact test *P*-value: 1.09 × 10^–40^) (Fig. 6a). The absence of major ZGA genes in the T247 group indicates that the observed HDACi effects on major ZGA can be fully explained by the action of MGCD0103. Among the genes affected by MGCD0103, the proportion of major ZGA genes that were downregulated was significantly higher than that of genes that were upregulated, i.e., 631 out of 2324 genes and 109 out of 1132 genes, respectively (Fisher’s exact test *P*-value: 9.83 × 10^–34^), suggesting that HDACi targeting Class I HDACs affect pre-implantation embryo development by suppressing the expression of major ZGA genes, which are normally upregulated during the 2-cell stage.

Next, to identify the biological processes that were affected by these HDACi-regulated major ZGA genes, we determined the enriched GO terms associated with major ZGA genes showing high differential expression, and labeled the evidence codes for the top 20 genes most affected by HDACi (Fig. 6b). The biological processes affected by HDACi targeting Class I HDACs mainly involved four aspects in major ZGA: RNA splicing, cyclin-dependent kinase activity, apoptotic signaling pathway, and transcription factor activity.

### HDACi treatment preferentially affected the expression of genes with specific epigenetic features

**Fig. 7:**
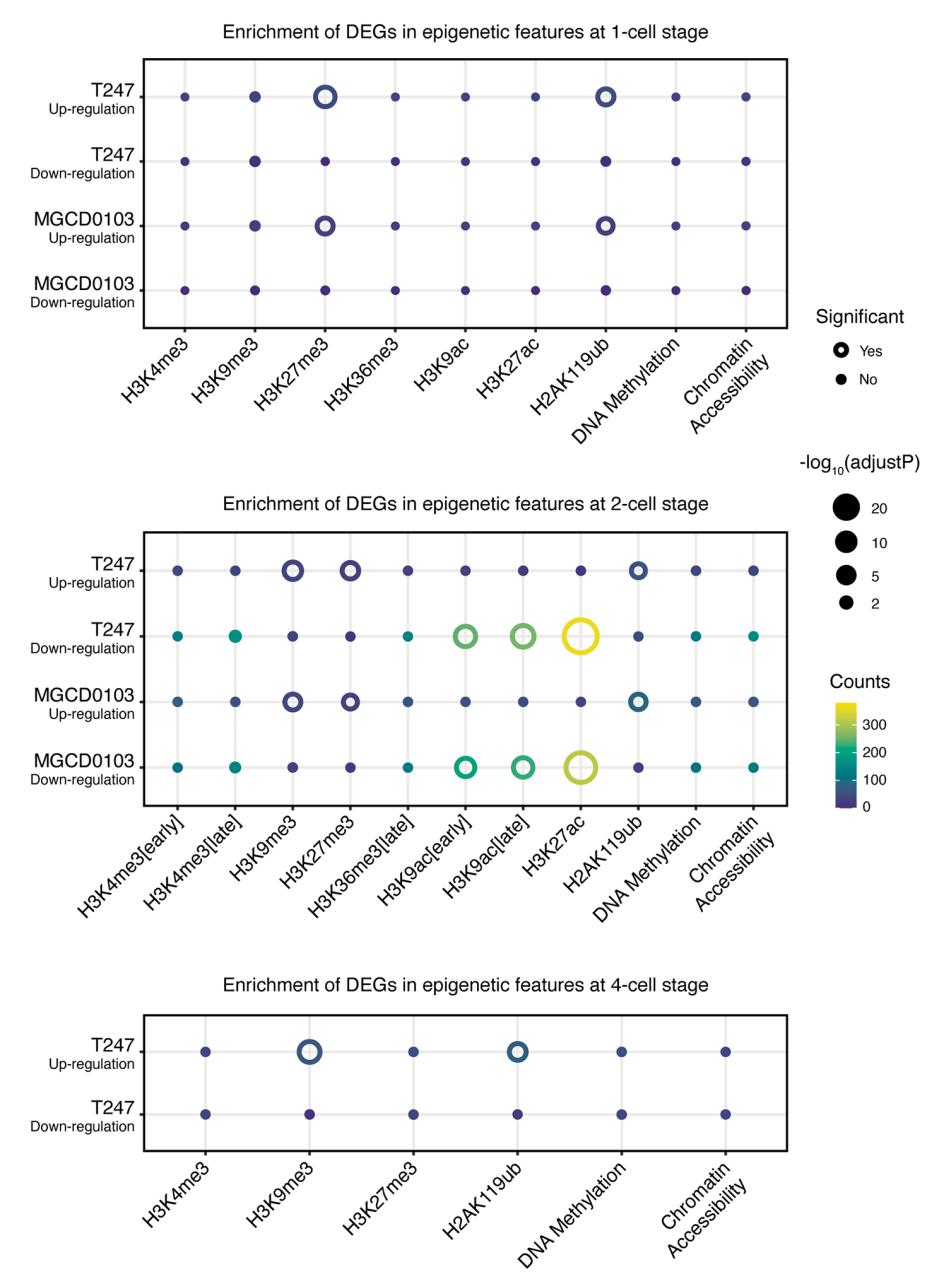
Relationship between the genes affected by HDACi and their epigenetic status. The dot heatmap shows the enrichment of DEGs that are up- or downregulated by the two HDACi at the 1-cell, 2-cell, and 4-cell stage, respectively, in the defined gene set, which exhibited high-level epigenetic modifications in the promoter region. The size of the dots/open circles (outer) represent the negative logarithm of the *P*-value adjusted for multiple testing in the hypergeometric test. An open circle denotes an adjusted *P*-value < 0.01, indicating a significant enrichment effect; otherwise, a point is used to indicate no significant enrichment. The color indicates the number of genes affected by HDACi in the defined high-level epigenetically modified gene set

In addition, to the functional analysis of DEGs, we analyzed the relationship between the DEGs and their epigenetic features. To illustrate the issue as comprehensively as possible, we collected data pertaining to histone modification, DNA methylation, and chromatin accessibility of these three stages of embryos from the NCBI Gene Expression Omnibus (GEO). We organized the sequencing data and defined the set of genes with strong epigenetic features in the promoter region. Subsequently, we compared it with the dataset of DEGs regulated by HDACi at each stage in the present experiment, and checked whether there was enrichment between them (Fig. 7). The HDACi-treated embryos exhibited an enrichment effect between the upregulated genes at the 1-cell stage and the highly modified genes of H3K27me3 and H2AK119ub. The enrichment effect observed between the upregulated genes on H3K27me3 and H2AK119ub persisted at the 2-cell stage and appeared on H3K9me3. A more substantial enrichment was detected among the downregulated genes with histone acetylation H3K9ac and H3K27ac. At the 4-cell stage, the enrichment effect corresponding to H3K27me3 in the upregulated genes disappeared, whereas H3K9me3 and H2AK119ub remained. This suggests that genes with certain histone modifications in their promoter region may be more susceptible to the effects of HDACi, with these modifications varying according to the embryonic stage. This phenomenon was attributed to HDAC3 rather than HDAC1/2, as it could also be observed when HDAC3 alone was inhibited.

## Discussion

The present study aimed to investigate the effect of selective HDACi treatment on pre-implantation embryos at the transcriptome level. The results showed that the effects of HDACi treatment on zygotes mainly occurred at the 2-cell stage; the T247 delayed early embryo development, whereas the MGCD0103 treatment prevented embryos to proceed into the 4-cell stage. Further analysis enabled the identification of the specific regulatory targets of the embryonic development triggered by these two HDACi. Moreover, we generated a profile of the biological processes that are affected by HDACi, and investigated the role of HDACi in two stages of ZGA. We inferred the potential main pathways via which HDACs affect gene expression in early embryos based on the transcriptional regulation and epigenetic features of the genes affected by HDACi. Finally, we observed a distinctive response of Hdac7 compared with other HDAC-family genes in early embryos after HDAC inhibition.

Previous studies have shown that treatment with pan-HDACi during the zygote stage inhibits embryonic development and causes arrest at the 2-cell stage [23, 25], which is consistent with the results obtained here using the Class I HDACi MGCD0103. In the PPI analysis, we identified Cdc25c (CDC25C), Cdk1 (CDK1), and Ccnb1 (cyclin B1) as hub genes that were affected by MGCD0103 in the cell cycle/division related protein network, with Cdc25c and Cdk1 being significantly downregulated. CDC25C and the CDK1/cyclin B1 complex are the main components of the G2/M DNA damage checkpoint, and their inactivation is a hallmark of cell arrest at the G2/M phase [35, 36]. Therefore, our findings suggest that treatment with MGCD0103 causes an arrest of early embryos at the 2-cell stage by affecting the G2/M DNA damage checkpoint, which is similar to its effect in various cancer cells [26, 37, 38]. Conversely, treatment with the HDAC3-specific inhibitor T247 only yielded a delay in embryonic development at the transcriptional level. Depletion of HDAC3 is typically believed to affect normal DNA repair during the S phase [39, 40]. A previous experiment localized HDAC3 to the mitotic spindle and demonstrated that its absence leads to defects in this structure [41]. Here, we observed a significant enrichment of genes related to mitotic-spindle organization among the DEGs after T247 treatment, and the hub genes identified in the cell-cycle-related protein network that was affected by T247 were mostly spindle-related genes. This suggests that the cell-development delay caused by the HDAC3-specific inhibitor T247 may be attributed to the impairment of the formation of a functional mitotic spindle. The comparison of the results obtained for the two HDACi allowed us to identify the main reason for the developmental arrest of early embryos at the 2-cell stage induced by the HDACi, i.e., the functional restriction of HDAC1/2, rather than HDAC3.

Our results also indicated that the effects of HDACi treatment on zygotes were most pronounced at the 2-cell stage, with a focus on specific biological processes, such as DNA repair [42], the cell cycle [37, 38, 43], apoptosis [44], energy metabolism [45], and mRNA processing [46], as reported previously in cancer cells. This highlights the critical period of the action of HDACs in early embryonic development, as the embryo undergoes ZGA during this period, thus marking the transition from maternal gene expression to zygotic gene expression [19, 21, 47]. As described in the results, the overall functional effect of HDACi on minor ZGA was difficult to observe. Therefore, our study aimed to identify key genes at minor ZGA stage. Among them, Gadd45g, which was specifically downregulated by MGCD0103, plays a role as an inhibitor of CDK1/cyclin B1 kinase during the G2/M DNA damage checkpoint [48], which suggests that the initial site of action of MGCD0103 treatment in cell-cycle-related processes. Contrary to minor ZGA, HDACi mainly downregulated the genes that were activated in the process of major ZGA, which was consistent with previous research [22, 25]. Our results further indicated that inhibition of HDAC1/2, rather than HDAC3, is the main cause of the activation of downregulated genes during major ZGA; we summarized the four biological processes that are affected by HDACi during major ZGA in Fig. 6b. Similar to the experiment using an HDAC1/2 dominant-negative mutant [25], embryos treated with MGCD0103 also exhibited downregulation of Kdm5b, but were not sensitive to other functional genes that are presumed to regulate ZGA in the mouse, such as Smarca4 (Brg1) and Nfya. This suggests that MGCD0103 treatment has a similar effect on the H3K4me3 histone modification state to that of the HDAC1/2 mutant during the regulation of the ZGA process, whereas it has a unique effect on the expression of several key genes.

Concomitantly, we also observed various unique phenomena triggered by the T247 treatment. For example, in our results, STAT3 was predicted as a potential transcriptional regulator among the genes that were upregulated by the HDAC3-specific inhibitor T247 at both the 1-cell and 2-cell stages, but was canceled in the 4-cell stage, which was not observed for MGCD0103. Previous studies have shown that HDAC3, rather than HDAC1/2, is the main enzyme that can deacetylate STAT3 to suppress the transcription of target genes [49]. This suggests that the HDAC3-specific inhibitor T247 may activate target genes not only by regulating the histone acetylation status, but also by removing non-histone acetylation states, such as STAT3, in early embryos.

Here, we also specifically focused on the unique effect of Hdac7 in the early embryonic response to HDACi. HDAC7 belongs to Class IIa HDACs and its knockout in mice leads to embryonic lethality [50]. Despite the controversy surrounding whether Class IIa HDACs can induce histone acetylation [11], HDAC7 was initially reported as a type of HDAC in mice [51], and the histone deacetylase activity of HDAC7 appears to be dependent on its interaction with HDAC3 [52]. Our results indicate that Hdac7 exhibited sustained upregulation in early embryos with Class I HDAC inhibition, unlike other HDAC-family genes that exhibit no expression differences or cell-stage-specific expression differences. This reflects the unique role of Hdac7 in the response to Class I HDAC inhibition. Because this phenomenon also occurred when HDAC3 alone was inhibited, it may suggest the existence of a compensatory mechanism of HDAC7 when it fails to obtain histone deacetylase activity by HDAC3. Interestingly, in cancer stem cells treated with MGCD0103, HDAC7 was significantly downregulated at the protein level [53], which may suggest a unique feedback mechanism of Hdac7 in early embryos that differs from that observed in cancer cells. Hdac7 has been extensively studied in immune-related cells, with critical roles in T- and B-cell development, lymphocyte activation, and inflammation [54]. However, there is a lack of research on Hdac7 in early embryonic development. Our experiments indicated that the immune-related protein interaction of Hdac7 was present in the HDACi effect as early as the 1-cell stage In the HDACi effect across cell stages, Hdac7 was observed in the high-loading genes that were differentially expressed between the HDACi treatment and control groups; moreover, antigen presentation and T-cell-cytotoxicity-related biological processes were significantly enriched. This suggests an effect of HDAC3 inhibition on immune-related biological processes, which is not affected by changes in early embryonic stages, possibly related to the upregulation of HDAC7.

The polycomb repressive complex (PRC) plays a role in the epigenetic regulation of transcription by modifying histones to silence target genes [55]. The PRC can be classified into two types; PRC1 induces the ubiquitination of the lysine 119 residue on histone H2A [56], whereas PRC2 catalyzes the mono-, di-, and tri-methylation of the lysine 27 residue on histone H3 [57]. The connection between the H2A119ub and H3K27me3 histone modifications can be established through the recruitment of PRC1 to chromatin via the binding of the classic PRC1 component CBX protein to the histone mark H3K27me3 [58], and H2AK119ub1 can guides zygotic deposition of H3K27me3 in mouse early embryos [59]. It has been demonstrated in multiple experiments that HDACs participate in the process of histone modification that is involved in polycomb-protein-mediated silencing by interacting with EZH2, which is the main component of PRC2 [60–63]. Our experimental results showed that genes that were upregulated by both HDACi from the 1-cell to 2-cell stage exhibited significant enrichment in genes with high levels of histone H3K27me3 or H2A119ub modifications in their promoter region. This suggests that the regulation of gene expression by HDACi in early embryos may be achieved not only through changes in the histone acetylation status, but also through the modulation of histone methylation/ubiquitination associated with polycomb proteins by HDAC3 (Fig. S4). Furthermore, the genes that were downregulated by the two HDACi at the 2-cell stage showed a higher level of enrichment in genes with high levels of H3K9ac and H3K27ac histone acetylation in their promoter. This is consistent with previous results from a comparison of transcriptome downregulation data and H3K27ac dynamics after treatment with the pan-HDACi TSA, and is thought to be a secondary effect of HDACi [22]. However, the specific mechanism underlying this phenomenon remain unclear. In summary, based on our experimental findings, we hypothesize that in early embryos treated with HDACi, the upregulated DEGs in the 1-cell and 2-cell stages may activate genes through multiple mechanisms. These include maintaining chromatin acetylation states and influencing other epigenetic modification states via interactions with PRC. We interpret these as direct effects of HDACi (Fig. S4). While in the 2-cell stage, the mechanisms behind the downregulated DEGs are more complex and challenging to explain, and we currently categorize them as indirect effects. However, we have identified certain characteristics within these genes. For instance, functionally, most downregulated genes are associated with major ZGA and weaken activation of zygotic genes (in contrast, the majority of upregulated genes are maternal genes, indicative of maintaining the maternal status of the embryo); epigenetically, genes with higher acetylation modifications in their promoters are more likely to be downregulated after HDACi treatment.

This study analyzes the effects of HDACi on early embryos only at the transcriptome level. Therefore, in our forthcoming research, we plan to design experiments based on these transcriptional characteristics observed after selective HDACi treatment. Our intention is to integrate these findings with HDAC protein experimental data and associated epigenetic modification data, and expand the observation of embryos to the blastocyst stage to enhance the interpretability of timelapse analysis as a way to further explore the specific mechanisms of gene expression regulation involved in selective HDACi treatment.

## Conclusions

Our study provided new insights into the effect of HDACi on early embryonic development at the transcriptome level. Our findings suggest that HDACi have a unique effect during the first two cell cycles of the embryo and can significantly affect ZGA. Furthermore, our results highlighted the differential effect of various selective HDACi and identified specific biological pathways and molecular mechanisms that are affected by HDACi treatment in early embryos. Future studies may integrate epigenetic modification data affected by HDACi, to further elucidate the molecular mechanisms underlying the observed effects and explore the potential applications of HDACi in the field of reproductive medicine.

## Methods

### Mice

All animal experiments were approved by the animal ethics committee of Kyushu University and were performed in accordance with the ARRIVE guidelines [64] and the regulations for animal experiments at Kyushu University. All mice were obtained from Charles River Laboratories Japan and were housed in individually ventilated cages under specific pathogen-free conditions. The animals were maintained on a 12-h light/dark cycle and at a room temperature of 23 to 25 °C and a humidity of 40–60%.

### In vitro fertilization

MII stage oocytes were collected from the oviducts of 8- to 9-week-old B6D2F1 female mice that had been treated with 7.5 IU pregnant mare serum gonadotropin and 7.5 IU human chorionic gonadotropin (ASKA Pharmaceutical). Subsequently, spermatozoa were collected from the cauda epididymis of 15- to 20-week-old ICR male mice. For in vitro fertilization (IVF), MII stage oocytes were placed in human tubal fluid (HTF) medium (Irvine Scientific) supplemented with bovine serum albumin (BSA, Sigma-Aldrich) at 10 mg/ml and were exposed to spermatozoa in which capacitation had been induced by prior incubation for 1 h under a humidified atmosphere of 5% CO_2_, 5% O_2_, and 90% N_2_ at 37 °C in HTF medium supplemented with BSA at 4 mg/ml. At 4 h post insemination (hpi), fertilized oocytes with two pronuclei were subjected to spermatozoa removal using a narrow-bore glass pipette, followed by culturing under a humidified atmosphere of 5% CO_2_, 5% O_2_, and 90% N_2_ at 37 °C in HTF medium containing BSA at 4 mg/ml; they were then covered with liquid paraffin (Wako).

### HDAC inhibition assay

Four hours after insemination by IVF, as described above, followed by the removal of surrounding spermatozoa from the fertilized oocytes using a narrow-bore glass pipette, zygotes were cultured for 6 h in HTF medium supplemented with BSA at 4 mg/ml and 10 µM MGCD0103 (Selleck Chemicals) or T247 [27], which are inhibitors of HDACs. The zygotes treated with HDACi were further cultured under the same conditions as the normal zygotes.

### Microarray data acquisition

The zygotes obtained after IVF were collected at 12, 24, and 48 hpi, and categorized as 1-cell stage, 2-cell stage, and 4-cell stage embryos, respectively. Total RNA was isolated and purified from 100 embryos using a PicoPure RNA Isolation Kit (Arcturus). cRNA was amplified and labeled using a Low input Quick Amp Labeling Kit (Agilent Technologies) and hybridized to a 60 K 60-mer oligomicroarray (SurePrint G3 Mouse Gene Expression Microarray 8 × 60 K; Agilent Technologies), according to the manufacturer’s instructions. The hybridized microarray slides were scanned using an Agilent scanner. The scanned images were processed using the Feature Extraction Software (version 9.5.1.1, Agilent Technologies), to calculate the relative hybridization intensity and background hybridization values using default parameters.

### Data processing

The raw signal intensities and flags for each probe were calculated from the hybridization intensities and spot information according to the procedures recommended by Agilent Technologies. Only spots with a signal flagged as “positive and significant” were used in the analysis. The remaining spots were processed as missing values. Probes with at least one valid value in three biological replicates were considered as valid detections. Probes that were valid detections in each condition were retained. Based on the evaluation criteria used in a previous study of microarray data [65], we evaluated the effectiveness of various missing value imputation methods on this experimental data (not shown), and ultimately selected the robust imputation method [66] to fill-in missing values. The probes were annotated using the annotation file (releases 2020.09.16) supplied by Agilent. In cases in which one gene corresponded to multiple probes, that with the highest signal intensity was selected as the representative probe. The signal intensity transformed by log2 was used in the subsequent analysis. Next, we used the quantro R package (version 1.32.0) [67] to evaluate global differences and detected a statistically significant change across condition groups (*P*-value < 0.01, 10 000 simulations). Therefore, smooth quantile normalization [68] was utilized for data normalization while preventing global modification.

### Principal component analysis

We performed a PCA of the normalized data using the prcomp function in R (version 4.2.0) [69], and generated a scree plot to determine the optimal number of principal components for retention. A 3D PCA plot was created using the scatterplot3d R package (version 0.3–44) [70].

### Differentially expressed genes

We used the normalized data as input for the limma R package (version 3.54.2) [71], and applied the least squares method to fit a linear model. Subsequently, we calculated the DEGs between the two conditions in all pairwise comparisons. The threshold for DEGs was set as|log2(FoldChange)| ≥1 and adjusted *P* < 0.05.

### Enrichment analysis

We utilized the clusterProfiler R package (version 4.6.2) [72] for enrichment analysis, and selected all terms from the GO database [73] as enrichment targets. Specifically, for the ordered set of all genes (e.g., based on the ranking of principal component loading), we performed a GSEA [74] and considered GO terms with an adjusted *P*-value < 0.05 and a|normalized enrichment score (NES)| ≥1 as being significantly enriched. For the specific gene sets (e.g., the DEG sets), we performed a statistical enrichment analysis and considered GO terms with an adjusted *P*-value < 0.05 as being significantly enriched. The redundancy was removed from the obtained enrichment GO term using the REVIGO online tools [75].

### Cluster analysis of DEGs

Before performing the cluster analysis, we utilized the factoextra R package (version 1.0.7) [76] to compute the Hopkins statistic. Data with a Hopkins statistic value < 0.75 were considered to exhibit a uniform distribution or lack statistically significant clusters. The optimal number of clusters was determined using the elbow method, which involves computing the within sum of squares, and were further validated using the gap statistic method. Subsequently, hierarchical clustering was performed using the ward.D2 method from the hclust function in R (version 4.2.0) [69], and the DEG expression data were grouped according to the determined optimal number of clusters.

### Prediction of transcription factors

We employed the iRegulon app (version 1.3) [77] with default parameters to predict TFs that are potentially involved in the upregulation of genes under the effects of HDACi (the threshold was set at NES > 3).

### Protein–protein interaction network construction and module analysis

Using the Cytoscape software (version 3.9.1) [78], we accessed and utilized the STRING database resource [79] to establish large-scale PPI networks based on the physical and functional associations between the proteins corresponding to the DEGs. Subsequently, we employed the MCODE plugin [80] with default parameters to identify potential functional modules (i.e., complexes of proteins that may share similar functions or participate in common biological processes) from the PPI network. The network graphs of modules were generated using the Cytoscape software (version 3.9.1) [78].

### Identification of hub genes

We employed cytoHubba [81], a plugin in the Cytoscape software (version 3.9.1) [78], to explore hub genes within the modules. Specifically, we used four local-based methods (Degree, MNC, DMNC, and MCC) to evaluate the importance of the nodes represented by each gene in the biological network. These methods generated rankings, which were then integrated using the RobustRankAggreg R package (version 1.2.1) [82], to obtain a comprehensive ranking of gene importance. Finally, the top two ranked genes were considered as hub genes within the modules.

### Visualization of the GO terms network

We identified numerous enriched GO terms among the thousands of DEGs assessed here, even after the removal of redundancy. It was challenging to intuitively understand these remaining GO terms. Therefore, we developed a visualization network that provided a comprehensive and intuitive representation of the relationships among the enriched biological processes. Specifically, as the input in this analysis, we used the biological process section of the enriched results after redundancy reduction. We calculated the Jaccard coefficient to represent the relationships between two enriched GO terms, and all the resulting Jaccard coefficients were then aggregated into an adjacency matrix, which served as the input for cluster analysis. We then assessed the clustering potential, inferred the optimal number of clusters, and performed clustering on the adjacency matrix data using the K-median method. Clusters with an average Jaccard coefficient < 0.05 were merged and labeled as “not categorized.” Subsequently, based on the adjacency matrix, we constructed network data using the GO terms as nodes and the Jaccard coefficients as weighted edges. We applied the Fruchterman–Reingold layout algorithm to compute the layout of the network, and removed outlier nodes from the layout. Finally, using the igraph R package (version 1.4.3) [83], we visualized the network data. In this visualization, the size of the nodes was controlled by the negative logarithm of the *P*-value in the enrichment analysis and the color of the nodes was determined by the clustering groups, whereas the thickness of the edges was controlled by the strength of the association between the GO terms. Furthermore, when one GO term was a subset of another, the edges were marked in brown, as Jaccard coefficients are not able to fully capture the concept of complete set inclusion.

### Annotation of GO-term clusters

We primarily utilized the tidytext R package (version 0.4.1) [84] for text mining and term frequency calculation to annotate the clusters of GO terms. Specifically, we downloaded the complete set of GO-term data (releases 2022.10.07) [73], and used the “definitions” of each GO term in the obo file as the corpus. We applied the TF-IDF algorithm to calculate the representative vocabulary for each GO term. Subsequently, in combination with the N-gram model, we performed term frequency calculations on all GO names within each GO-term cluster. The final list of words/phrases was re-scored based on three features: (1) the term frequency, (2) the lowest *P*-value associated with their occurrence in the GO term name, and (3) whether they were representative terms for that GO term. For each GO-term cluster, the most suitable annotation was selected from the top three candidates with the highest scores.

### Classification of pre-implantation embryonic genes

We classified the genes based on the programmed waves of pre-implantation embryos [19]. Specifically, we referenced previous research [85] and employed the fuzzy c-means clustering algorithm from the Mfuzz R package (version 2.58.0) [86] to perform soft clustering (which is suitable for analyzing temporal microarray data) on the data of MII oocytes, zygotes, 2-cell and 4-cell stage embryos. According to the expression patterns observed in the clustering results (Fig. S5), the genes in clusters 6, 9, 10, 17, 22, and 23 were defined as “maternal genes”; the genes in clusters 5, 12, 14, and 24 were defined as “minor ZGA genes”; the genes in clusters 1, 15, and 25 were defined as “major ZGA genes”; the genes in clusters 7, 13, 19, and 21 were defined as “MGA (mid-preimplantation gene activation) genes”; and the genes in the remaining clusters were labeled as “other genes.”

### Association analysis of DEGs and their inherent epigenetic modification features

The histone modification data (H3K4me3 [87] from GSE71434, H3K9me3 [88] from GSE98149, H3K27me3 [89, 90] from GSE76687 and GSE73952, H3K36me3 [91] from GSE112834, H3K9ac [92] from GSE143523, H3K27ac [22, 93] from GSE72784 and GSE207222, and H2AK119ub [94] from GSE153531), chromatin accessibility and DNA methylation data [95] from GSE136718 were processed separately according to the data source paper workflow, to obtain BED files. Because the subsequent analyses relied solely on the ranking of genes within each dataset, no normalization correction was applied to the histone modification data. The data were annotated using GRCm39 (musculus annotation release 104) [96]. The promoter region of histone modification data was uniformly defined as TSS −/+1 kb, whereas the definitions of the original paper were retained for chromatin accessibility data and DNA methylation data, i.e., TSS − 200 b/+100 b and − 1 kb/+0.5 kb, respectively. The scores within the promoter region of each gene were summed to obtain the corresponding feature intensity. One thousand genes with the highest intensity in each data were defined as genes with a strong association of the feature (the number of genes defined according to this criterion was not strict, as we also reached similar conclusions using definitions based on 500 or 2000 genes). We ran a hypergeometric test between DEGs and the gene set that was strongly associated with the feature under the same embryo stage. Finally, adjusted *P* < 0.01 was used as a threshold to determine whether there was an enrichment effect between the DEGs and the corresponding feature gene sets.

### Statistical analysis and visualization

All statistical calculations were performed in R (version 4.2.0) [69]. The *P*-value was adjusted using the Benjamini–Hochberg method in multiple hypothesis tests, and was recorded as adjusted *P*.

If not specified otherwise, all images were generated using the ggplot2 R package (version 3.4.2) [97].

### Electronic supplementary material

Below is the link to the electronic supplementary material.

Supplementary Material 1

Supplementary Material 2

Supplementary Material 3

Supplementary Material 4

Supplementary Material 5

Supplementary Material 6

Supplementary Material 7

Supplementary Material 8

Supplementary Material 9

Supplementary Material 10

Supplementary Material 11

Supplementary Material 12

Supplementary Material 13

## Data Availability

The datasets generated during the current study are available in the GEO repository, the accession number GSE238124. The datasets supporting the conclusions of this article are included within the article and its additional files. Further information and requests for datasets and scripts generated in the present study should be directed to and will be fulfilled by the Lead Contact, Mikita Suyama (mikita@bioreg.kyushu-u.ac.jp).

## Abbreviations

BSA: Bovine serum albumin
DEG: Differentially expressed gene
GEO: Gene expression omnibus
GO: Gene ontology
GSEA: Gene set enrichment analysis
GV: Germinal vesicle
HAT: Histone acetyltransferase
HDAC: Histone deacetylase
HDACi: HDACs inhibitor
hpi: Hour post insemination
HTF: Human tubal fluid
IVF: In vitro fertilization
MGA: Mid-preimplantation gene activation
MII: Metaphase II
MZT: Maternal-to-zygotic transition
NES: Normalized enrichment score
PCA: Principal component analysis
PPI: Protein–protein interaction
PRC: Polycomb repressive complex
TF: Transcription factor
ZGA: Zygotic gene activation
