# Supplementary material for: The impact of selective HDAC inhibitors on the transcriptome of early mouse embryos
Source: BMC Genomics. 2024 Feb 5;25:143. doi: 10.1186/s12864-024-10029-3 (PMC10840191; doi:10.1186/s12864-024-10029-3)
Supplement: Supplementary file 1 — Supplementary Material 1 [file 12864_2024_10029_MOESM1_ESM.pdf]

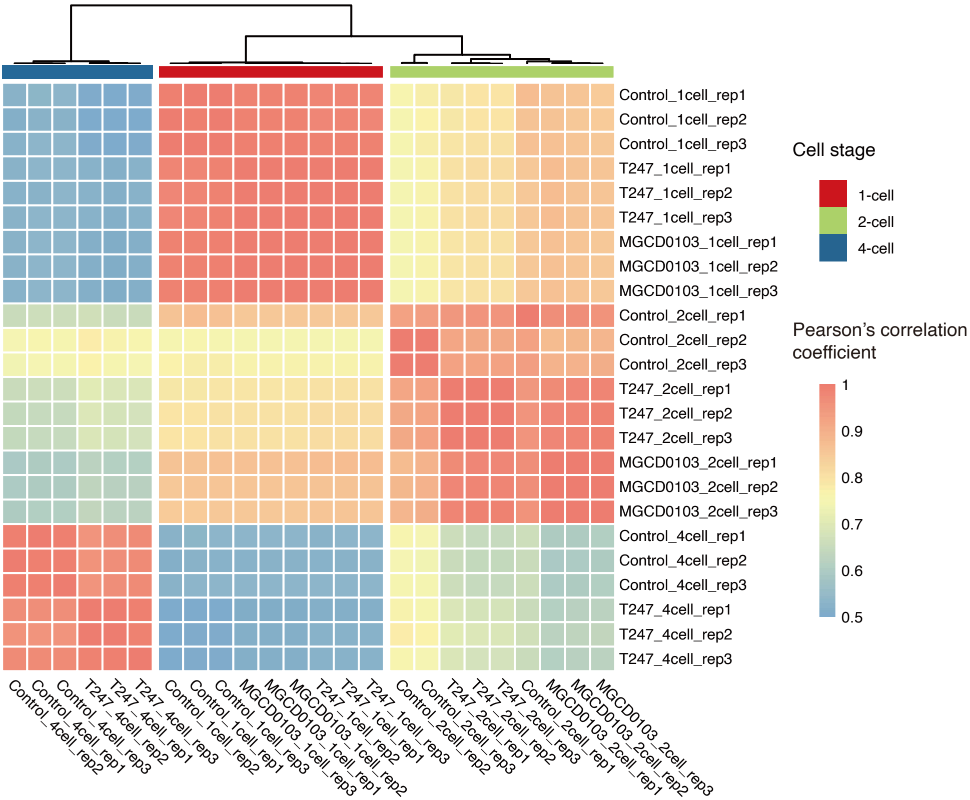

Fig. S1. Correlation of all samples. The heatmap shows the correlations between all pairs of samples. Hierarchical clustering was performed only in the vertical direction, and different cell stage data were annotated with different colors below the clustering tree.
