# Supplementary material for: The impact of selective HDAC inhibitors on the transcriptome of early mouse embryos
Source: BMC Genomics. 2024 Feb 5;25:143. doi: 10.1186/s12864-024-10029-3 (PMC10840191; doi:10.1186/s12864-024-10029-3)
Supplement: Supplementary file 2 — Supplementary Material 2 [file 12864_2024_10029_MOESM2_ESM.pdf]

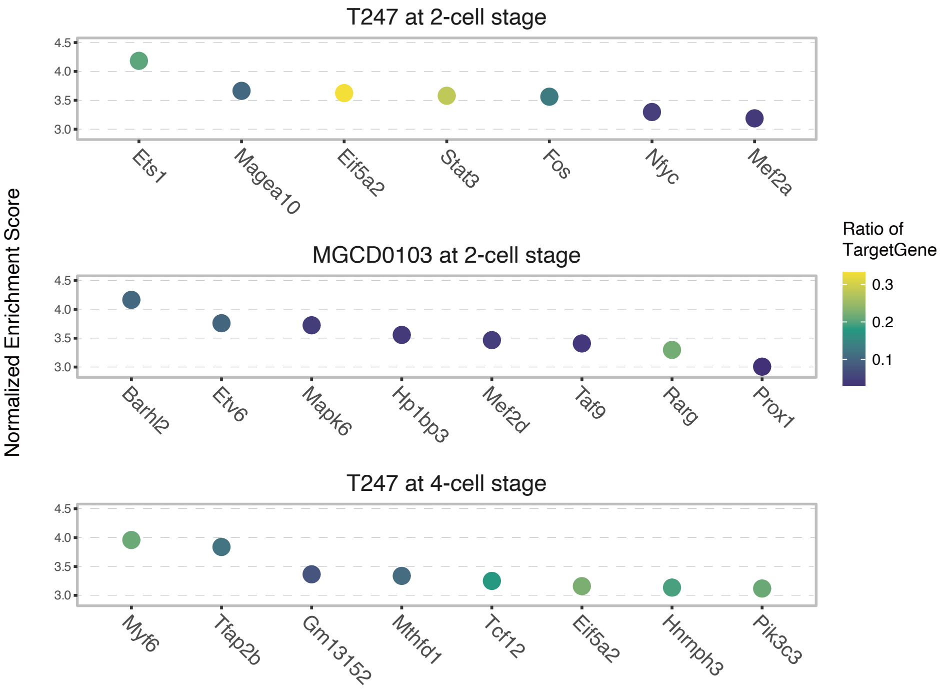


**Fig. S2. TFs preddicted to regulate DEGs.** The plot shows results of predicted TFs that regulate DEGs at 2-cell and 4-cell stage using the same display approach as in figure 2c.
