# Supplementary material for: The impact of selective HDAC inhibitors on the transcriptome of early mouse embryos
Source: BMC Genomics. 2024 Feb 5;25:143. doi: 10.1186/s12864-024-10029-3 (PMC10840191; doi:10.1186/s12864-024-10029-3)
Supplement: Supplementary file 3 — Supplementary Material 3 [file 12864_2024_10029_MOESM3_ESM.pdf]

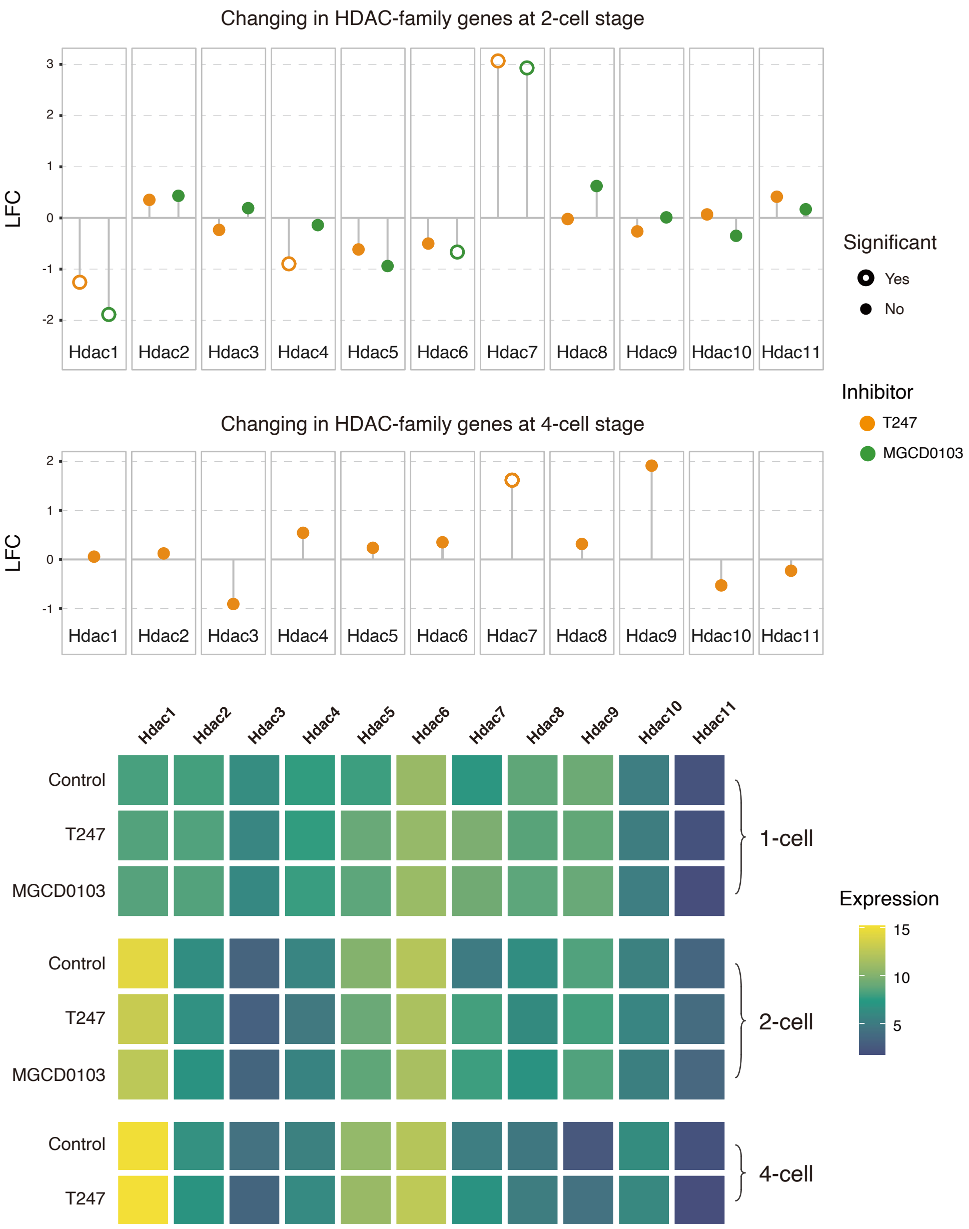

Fig. S3. Expression of HDAC-family genes after HDACi treatment. The lollipop plots above the figure show differential expression results of HDAC-family genes after HDACi treatment at 2-cell and 4-cell stage using the same display approach as in figure 2f. The heatmap below the figure shows the absolute expression of HDAC-family genes after HDACi treatment at the 1-cell, 2-cell, and 4-cell stages.
