# Supplementary material for: The impact of selective HDAC inhibitors on the transcriptome of early mouse embryos
Source: BMC Genomics. 2024 Feb 5;25:143. doi: 10.1186/s12864-024-10029-3 (PMC10840191; doi:10.1186/s12864-024-10029-3)
Supplement: Supplementary file 4 — Supplementary Material 4 [file 12864_2024_10029_MOESM4_ESM.pdf]

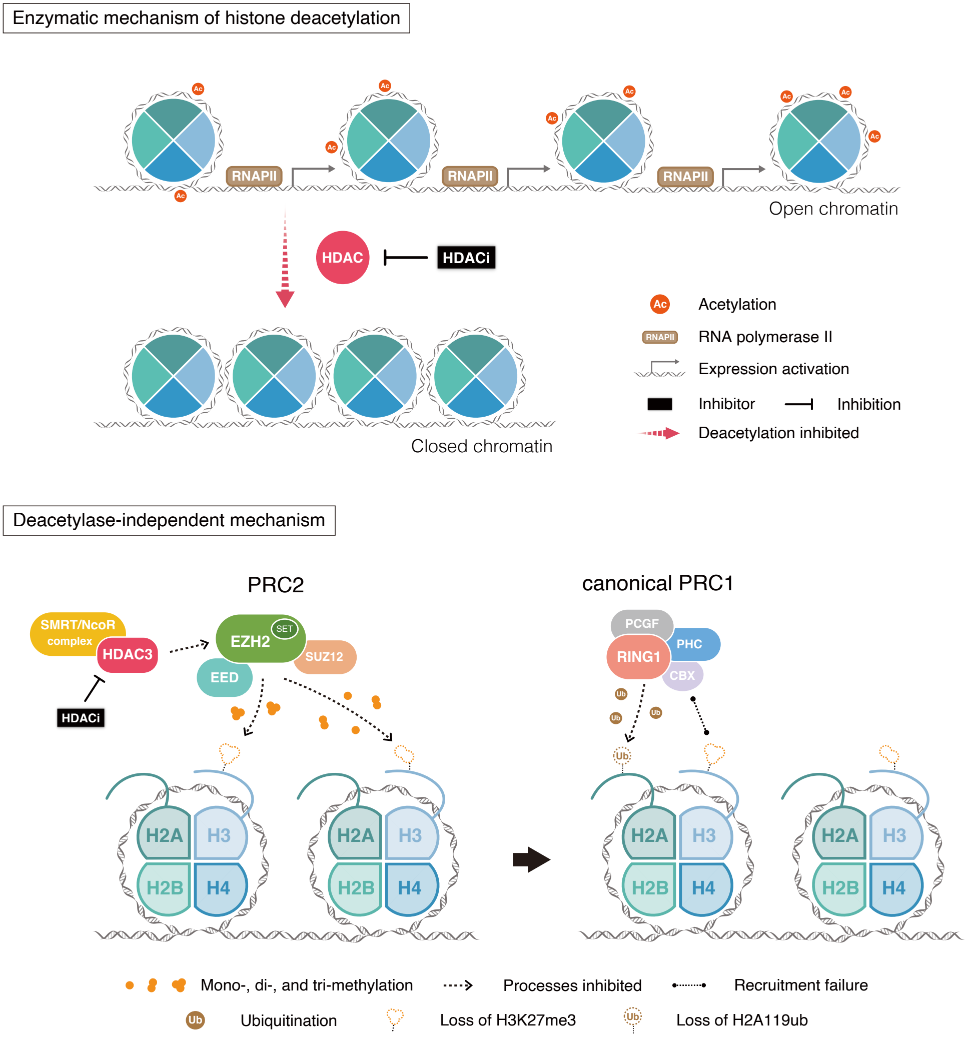

Fig. S4. Potential mechanisms of gene upregulation in early embryos after HDACi treatment. The figure shows the potential mechanisms of gene activation in early embryos due to the direct effects of HDACi. The first mechanism involves HDACi maintaining an open chromatin state by inhibiting the deacetylase activity of HDACs, thereby activating gene expression. The second mechanism operates through the prevention of the non-enzymatic recruitment of PRC2 by HDAC3, leading to a reduction in the deposition of the gene silencing markers H3K27me3 and H2A119ub at gene promoters, consequently upregulating gene expression. SMRT, silencing mediator of retinoic acid and thyroid hormone receptor; NCoR, nuclear receptor co-repressor 1; EZH2, enhancer of zeste homologue 2; EED, embryonic ectoderm development protein; SUZ12, suppressor of zeste 12 homologue; RING1, really interesting new gene 1 finger protein, functioning here as an E3 ubiquitin ligase; CBX, chromobox protein; PHC, polyhomeotic protein; PCGF, polycomb group RING finger protein.
