# Supplementary material for: The impact of selective HDAC inhibitors on the transcriptome of early mouse embryos
Source: BMC Genomics. 2024 Feb 5;25:143. doi: 10.1186/s12864-024-10029-3 (PMC10840191; doi:10.1186/s12864-024-10029-3)
Supplement: Supplementary file 5 — Supplementary Material 5 [file 12864_2024_10029_MOESM5_ESM.pdf]

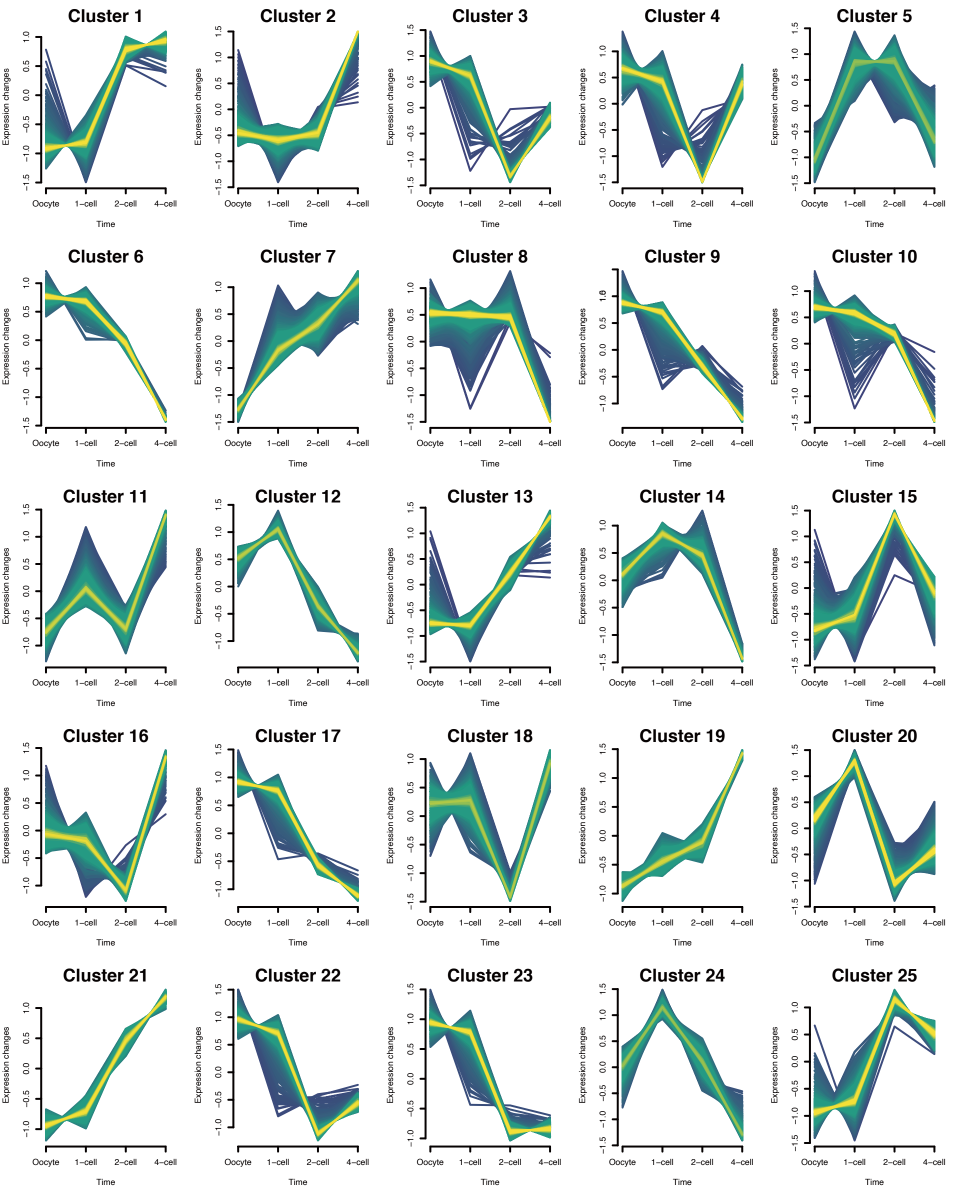

Fig. S5. Results of Mfuzz analysis of temporal gene expression patterns. The line plots show the trend of temporal expression changes in the four stages before and after fertilization, which are then divided into 25 different clusters.
